# Induction, Flavonoids Contents, and Bioactivities Analysis of Hairy Roots and True Roots of *Tetrastigma hemsleyanum* Diels et Gilg

**DOI:** 10.3390/molecules28062686

**Published:** 2023-03-16

**Authors:** Hongzhen Wang, Anran Wang, Hanying Pu, Yuxin Yang, Zeyuan Ling, Haishun Xu, Juan Xu, Haizheng Yu, Xueqian Wu

**Affiliations:** 1 State Key Laboratory of Subtropical Silviculture, Department of Chinese Herbal Medicine Zhejiang Agriculture and Forestry University, Hangzhou 311300, China; 2 Zhejiang Provincial Key Laboratory of Resources Protection and Innovation of Traditional Chinese Medicine, Department of Chinese Herbal Medicine Zhejiang Agriculture and Forestry University, Hangzhou 311300, China; 3 Zhejiang Wuyangtang Chinese Medicine Group Co., Ltd., Lishui 323300, China

**Keywords:** *Tetrastigma hemsleyanum* Diels et Gilg, hairy roots, *Agrobacterium rhizogenes* strain Ar Qual, (+)-Catechin, (−)-Epicatechin

## Abstract

The flavonoids in *Tetrastigma hemsleyanum* Diels et Gilg (*T. hemsleyanum*) have high medicinal value. However, because of slow growth and harsh ecological environments, *T. hemsleyanum* is currently an endangered species. In light of this, we present a detailed hairy root induction procedure as a promising alternative to true roots with medicinal value. The percentage of explants induced by *Agrobacterium rhizogenes* (*A. rhizogenes*) to produce hairy roots out of the total number of explants infected (induction rate 1) was 95.83 ± 7.22%, and the proportion of hairy roots that contained *Rol* B fragments among all the hairy roots with or without *Rol* B fragments (positive rate) was 96.57 ± 1.72%. The transformation was further confirmed by the expression of the GUS protein. A high-productive hairy root line was screened for the comparative profiling of six flavonoids with true roots using high-performance liquid chromatography (HPLC). The contents of (+)-catechin, (−)-epicatechin, neochlorogenic acid, luteolin-6-C-glucoside, and orientin were 692.63 ± 127.24, 163.34 ± 31.86, 45.95 ± 3.46, 209.68 ± 6.03, and 56.82 ± 4.75 μg/g dry weight (DW) of 30-day-old hairy roots, respectively, which were higher than those of 3-year-old true roots. Hairy roots have stronger antioxidant activity than true roots. Overall, the hairy roots of *T. hemsleyanum* could serve as promising alternative sources for the production of flavonoids with medicinal uses.

## 1. Introduction

*Tetrastigma hemsleyanum* Diels et Gilg (*T. hemsleyanum*), of the grape family Vitaceae, is a functional food and traditional medicinal plant in China. Its true roots can be used to treat fevers, stimulate blood circulation, relieve pain, dispel wind, and reduce phlegm. It may also be used in detoxification [1]. *Tetrastigma hemsleyanum* also has antibacterial [2], antiviral [3], and anti-inflammatory properties [4]. The curative effect of *T. hemsleyanum* is closely related to the types and contents of flavonoids within it [5,6].

*Tetrastigma hemsleyanum* takes 3–5 years to form the true roots used in medicine. It grows under harsh environmental conditions such as within the crevices of rocks near valleys on hillsides, at a height of 300–1300 m [7]. While tissue culture and the artificial cultivation of *T. hemsleyanum* have been successful, the production cycle for the formation of true roots from a small plant is still very long [7]. Thus, *T. hemsleyanum* production hardly meets the increasing market demand [8]. In recent years, *T. hemsleyanum* has become an endangered and precious species in China due to its medicinal and economic value [9], boasting a market price of $58.25 per kilogram [1,10]. As such, the production and utilization issues associated with a long growth cycle and many ecological environment requirements need to be solved urgently.

Compared to the original plant, hairy roots are characterized by high contents of active components, biomass increase, biochemical and genetic stability, and easy operation and control; consequently, they have emerged as an alternative to traditional breeding strategies [11,12]. For instance, the total tanshinone content reached up to 15.4 mg/g dry weight (DW) in the hairy roots of transgenic *Salvia miltiorrhiza* that were further cultured on 1/2 MS liquid medium for 2 months, while the tanshinone content of the field-grown plant roots was only 1.7–9.7 mg/g DW [13]. More than 300 species of hairy roots have been established in the culture system and used to produce bioactive compounds. Of these, 76% are herbaceous plants, such as the sunflower *Helianthus annuus* L. [14], as well as *Urena lobata* [15] and *Centella asiatica* [16]. Because of the difficulty in infecting woody plants and lianas, woody plants such as *Taxus* × *media* have accounted for 17% [17], while lianas such as *Tripterygium wilfordii* have accounted for 7% [18].

*Tetrastigma hemsleyanum*, a type of perennial liana, has been reported to induce hairy roots via an *Agrobacterium rhizogenes* (*A. rhizogenes*)-mediated approach [7]. Specifically, hairy roots were induced using the cucumopine type *A. rhizogenes* K599, and the optimization of both the induction medium and co-culture medium was reported in this study. Although previous studies have generated valuable data on the genetic transformation system of *T. hemsleyanum*, there are no protocols or cultivation conditions that are appropriate for all genotypes because of the diversity in genomic sequences, explants, hormones, and required culture conditions [7,19]. Consequently, there is an urgent need to develop a biotechnological system for the production of flavonoids as an alternative to the *T. hemsleyanum* true roots. The existing literature on the *T. hemsleyanum* inducing system has primarily focused on the contents of total flavonoids, quercetin, and kaempferol [7]. The *T. hemsleyanum*-induced hairy root system for producing the primary bioactive components—specifically (+)-catechin, (−)-epicatechin, neochlorogenic acid, luteolin-6-C-glucoside, and orientin—has not been sufficiently documented. This study seeks to optimize the induction system for *T. hemsleyanum* hairy roots to address this gap in knowledge. Additionally, the antioxidant activity of the hairy roots and true roots of *T. hemsleyanum* will be used to assess the potential of hairy roots as an efficient alternative strategy for true roots of *T. hemsleyanum*.

## 2. Results

### 2.1. Induction of Hairy Roots

Leaves’ explants responded more to the induction of hairy roots than internodal explants when *A. rhizogenes* Ar Qual was inoculated into *T. hemsleyanum* to infect stems and leaves. The positive acquisition rate of leaves was 317.27 ± 57.10%, and the positive acquisition rate of stems was 25.71 ± 13.42% (Table 1). Therefore, leaf explants produced significantly more hairy roots than stem explants (*p* ≤ 0.05). No similar root induction was observed in the control explants. The infective mode of vacuuming can cause the inactivation of explant cells (Table 2). After infecting the leaf explants with *A. rhizogenes* C58C1 (OD600 = 0.5–0.6) and *A. rhizogenes* Ar Qual (OD600 = 0.5–0.6) to induce hairy roots, the induction rate 1 of *A. rhizogenes* Ar Qual (75.17 ± 7.42%) was higher than that of *A. rhizogenes* C58C1 (36.73 ± 4.02%) (Table 1). The positive acquisition rate of hairy roots induced by *A. rhizogenes* Ar Qual (317.27 ± 57.10%) was higher than that induced by *A. rhizogenes* C58C1 (40.00 ± 17.14%).

The hairy root induction by *A. rhizogenes* Ar Qual was further optimized by testing different co-cultivation times and induction medium types. Co-cultivation for 3 days showed the highest induction rate 1 (65.14 ± 14.83%) and induction rate 2 (326.65 ± 64.75%), followed by co-cultivation for 2 days (18.76 ± 5.59%) and induction rate 2 (82.14 ± 21.71%) (Table 3). After co-cultivation for 1 day, the induction effect was not obvious. After co-cultivation for 4 days, *A. rhizogenes* overgrew and produced toxic effects. Different medium types contained different nutrient elements, which could promote or inhibit hairy root induction. The induction effect of 1/4 MS nutrient medium supplemented with 1.0 mg/L IBA and 1.0 mg/L KT was the best (Table 4). The induction rates 1 and 2 of 1/4 MS medium were 95.83 ± 7.22% and 951.39 ± 231.85%, respectively. The induction rate 1 values of the MS and N6 media were only 25.00 ± 8.33% and 16.67 ± 5.56%, and their induction rate 2 values were only 52.78 ± 31.55% and 16.67 ± 5.56%, respectively.

**Figure 1:**
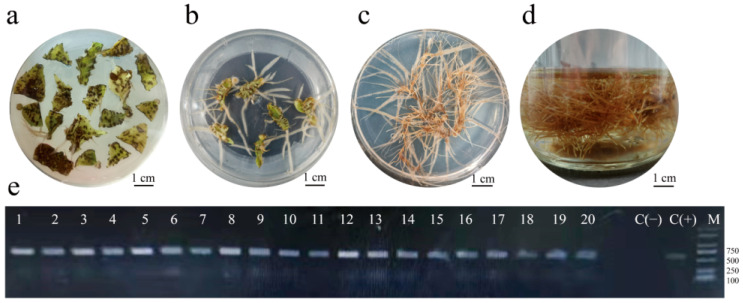
*A. rhizogenes* Ar Qual-mediated hairy roots culture. (**a**) Root bulges were formed after the infection of *A. rhizogenes* Ar Qual. (**b**) Hairy roots were formed after the infection of *A. rhizogenes* Ar Qual. (**c**) Solid culture of hairy roots. (**d**) Liquid culture of hairy roots. (**e**) PCR analysis of the *Rol* B gene in the hairy root lines of *T. hemsleyanum* [lane M, marker; L1–L20, the hairy roots lines induced by *A. rhizogenes* Ar Qual; C(+), plasmid DNA from *A. rhizogenes* Ar Qual (positive control); C(−), roots from a non-transformed control plant (negative control)].

The leaves were selected as explants, infected by *A. rhizogenes* Ar Qual, co-cultured on MS medium with 0.4 mg/L NAA and 19.62 mg/L AS for 3 days, and then transferred to 1/4 MS medium with 1.0 mg/L IBA and 1 mg/L KT for induction (pH 5.8). When the hairy root length reached 2 cm, the candidate hairy root lines were transferred to B5 medium with 1.0 mg/L IBA and 1.0 mg/L KT (pH 5.5) for solid multiplication or liquid multiplication, and the complete procedure is shown in Figure 1. After *A. rhizogenes* Ar Qual infected leaves’ explants, root bulges developed within 9 days (Figure 1a), with hairy roots developing within 15 days (Figure 1b). The whole process was completed in 45 days (Figure 1c,d) until the end of the massive proliferation of hairy roots. A PCR analysis using *Rol* B specific primer with the fragment of ~678 bp confirmed the transgenic roots. The fragment for *Rol* B was observed in the amplified DNA from all the 22 hairy root lines and the positive control (Figure 1e). No such amplification product was found in the DNA isolated from the negative control (Figure 1e).

**Figure 2:**
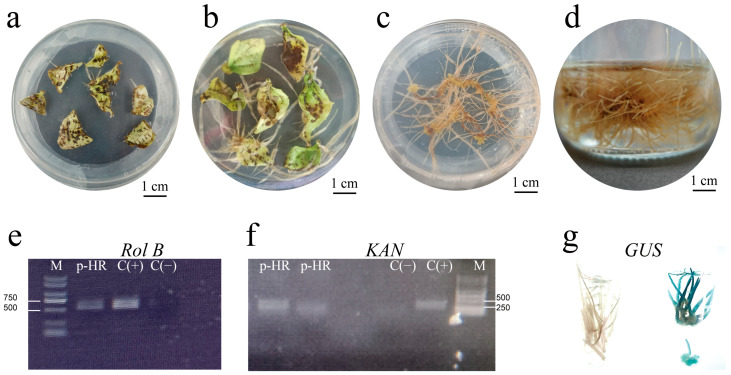
*A. rhizogenes* Ar Qual strain carrying pCAMBIA 1381Z-mediated hairy root culture. (**a**) Root bulges were formed after the infection of *A. rhizogenes* Ar Qual strain carrying pCAMBIA 1381Z. (**b**) Hairy roots were formed after the infection of *A. rhizogenes* Ar Qual strain carrying pCAMBIA 1381Z. (**c**) Solid culture of hairy roots. (**d**) Liquid culture of hairy roots. (**e**) PCR analysis of the *Rol* B gene in the hairy root line of *T. hemsleyanum* [lane M, marker; Lp-HR, the hairy roots lines induced by *A. rhizogenes* Ar Qual strain carrying pCAMBIA 1381Z; C(+), plasmid DNA from *A. rhizogenes* Ar Qual strain carrying pCAMBIA 1381Z (positive control); C(−), roots from a non-transformed control plant (negative control)]. (**f**) PCR analysis of the KAN gene in the hairy root lines of *T. hemsleyanum* [lane M, marker; Lp-HR, the hairy roots lines induced by *A. rhizogenes* Ar Qual strain carrying pCAMBIA 1381Z; C(+), plasmid DNA from *A. rhizogenes* Ar Qual strain carrying pCAMBIA 1381Z (positive control); C(−), roots from a non-transformed control plant (negative control)]. (**g**) GUS staining in hairy roots. HR, the hairy root lines induced by *A. rhizogenes* Ar Qual-wild type strain; p-HR, the hairy root lines induced by *A. rhizogenes* Ar Qual strain carrying pCAMBIA 1381Z.

Under the same conditions, *A. rhizogenes* Ar Qual harboring the binary vector pCAMBIA 1381Z (CAMBIA)—which contains GUS ORF and the selective marker gene kanamycin resistance (KAN) under the control of CaMV35S promoter—successfully infected the leaf to induce hairy roots for system stability and genetic transformation verification (Figure 2a–d). The PCR primers *Rol* B and KAN were used to identify hairy roots (Figure 2e,f). Meanwhile, blue color was observed on the hairy roots transformed with the Ar Qual strain carrying pCAMBIA 1381Z and no color was observed on the Ar Qual wild-type strain-transformed hairy roots (Figure 2g).

### 2.2. Selection of Hairy Root Lines with High Biomass and High Flavonoids Contents

**Figure 3:**
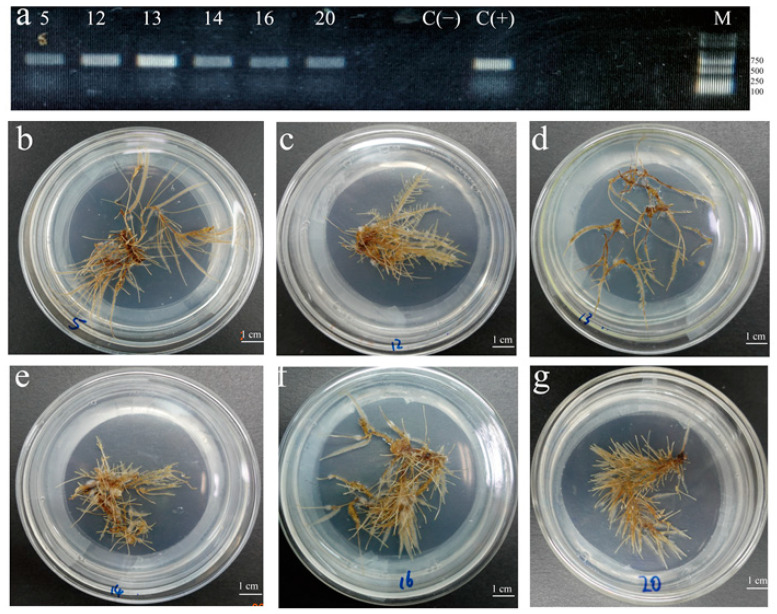
PCR analysis and phenotypes of 6 *T. hemsleyanum* hairy root lines. (**a**) PCR analysis of the *Rol* B gene in the hairy root line of *T. hemsleyanum* [lane M, marker; L1–L6, the hairy roots lines induced by *A. rhizogenes* Ar Qual, named HR4, 5, 12, 13, 14, 16, and 20; C (+), plasmid DNA from *A. rhizogenes* Ar Qual (positive control); C (−), roots from a non-transformed control plant (negative control)]. (**b**) HR5, (**c**) HR12, (**d**) HR13, (**e**) HR14, (**f**) HR16, and (**g**) HR20.

**Figure 4:**
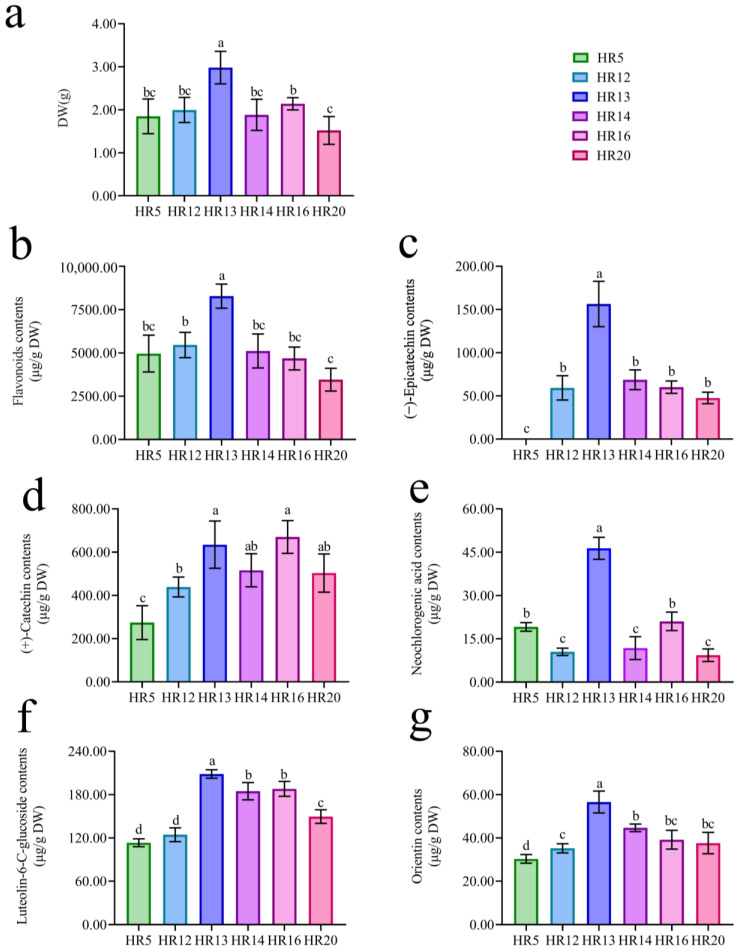
Evaluation of biomass productivity and flavonoids yield of *T. hemsleyanum* hairy roots lines. (**a**) Dry weight comparison of hairy roots lines. (**b**) Total flavonoids contents of hairy roots lines. (**c**) (−)-Epicatechin contents of hairy roots lines. (**d**) (+)-Catechin contents of hairy roots lines. (**e**) Neochlorogenic acid contents of hairy roots lines. (**f**) Luteolin-6-C-glucoside contents of hairy roots lines. (**g**) Orientin contents of hairy roots lines. All the experiments were performed in triplicate with at least three independent runs (*n* = 9). Significant differences are represented by different letters (a–d) (*p* < 0.05).

Six thriving hairy root lines containing *Rol* B were screened (Figure 3a). They were named HR5, 12, 13, 14, 16, and 20 that were cultured (Figure 3b–g). HR13 had the highest dry weight (2.89 ± 0.38 g), whereas HR20 had the lowest dry weight (1.52 ± 0.32 g) (Figure 4a). Significant differences were found between HR20 and HR13.

A regression calculation of total flavonoids concentration by absorbance was carried out, and the following linear equation was obtained—A = 9.6714C + 0.0251 R^2^ = 0.999, indicating that the mass concentration had a good linear relationship with the absorbance value in the range of 0–0.5 mg/mL. Among the different samples, HR13 was the highest flavonoids content (8278.27 ± 698.10 μg/g DW) and HR20 was the lowest flavonoids content (3453.05 ± 656.99 μg/g DW) (Figure 4b).

The standard curves of each standard substance, as determined by HPLC, were as follows (Table 5). According to R^2^, the standard curve has a good linear relationship in the range of the mass concentration of standard substance from 40.00 μg/mL to 240.00 μg/mL, from 0.30 μg/mL to 24.00 μg/mL, or from 1.23 μg/mL to 66.67 μg/mL. The limit of detection (LOD) and limit of quantitation (LOQ) were from 0.150 μg/mL to 0.025 μg/mL and from 0.300 μg/mL to 0.060 μg/mL, respectively, which could meet the needs of the quantitative detection of each target.

The (+)-catechin contents and (−)-epicatechin contents of different hairy root lines were determined (Supplementary Materials Figure S1). No significant differences in (+)-catechin contents were found between HR16 (670.02 ± 75.72 μg/g DW) and HR13 (634.46 ± 109.12 μg/g DW) (Figure 4d). HR13 was the highest (−)-epicatechin contents (156.29 ± 26.24 μg/g DW) (Figure 4c). Furthermore, HR13 was the highest neochlorogenic acid contents (46.34 ± 3.82 μg/g DW), luteolin-6-C-glucoside contents (208.48 ± 5.82 μg/g DW), and orientin contents (56.55 ± 5.05 μg/g DW) (Figure 4e–g, Supplementary Materials Figures S2 and S3). Therefore, HR13 was identified as a hairy root line for subsequent experiments.

### 2.3. Comparison of Flavonoids Content of Hairy Roots and True Roots

The total flavonoids content of hairy roots (8919.48 ± 740.97 μg/g DW) was approximately 1/2 of the total flavonoids content of true roots (19,851.63 ± 575.36 μg/g DW) (Table 6). Qualitative and quantitative analyses of target chemical composition in the hairy roots and true roots were performed (Supplementary Materials Figure S4). The (+)-catechin and (−)-epicatechin contents in the hairy roots were 692.63 ± 127.24 and 163.34 ± 31.86 μg/g DW, respectively, whereas those in the true roots were 622.52 ± 97.53 and 70.21 ± 25.12 μg/g DW, respectively (Table 6). The neochlorogenic acid, luteolin-6-C-glucoside, and orientin contents in the hairy roots were 45.95 ± 3.46, 209.68 ± 6.03, and 56.82 ± 4.75 μg/g DW, respectively, which were higher than those in the tuber roots. The neochlorogenic acid, luteolin-6-C-glucoside, and orientin contents in the true roots were15.23 ± 0.38, 185.29 ± 1.19, and 44.06 ± 0.79 μg/g DW, respectively. This showed that the accumulation of metabolites in the hairy roots and true roots was changed.

### 2.4. Comparison of Antioxidant Activity of Hairy Roots and True Roots

The DPPH free-radical scavenging activity assay was used to determine the antioxidant activity of the crude extract of *T. hemsleyanum* hairy roots, which laid a foundation for its potential application in food, health products, and medicine fields. The experimental results are shown in Table 7. The free-radical scavenging ability (IC50 = 1.41 ± 0.24 μg/mL) of (+)-catechin was the highest. The free-radical scavenging ability (IC50 = 1.64 ± 0.16 μg/mL) of the hairy root extract was significantly higher than that of the true root extract (IC50 = 2.34 ± 0.15 μg/mL).

## 3. Discussion

### 3.1. Establishment of a Highly Efficient Hairy Roots Induction System

Like other woody perennials, the Vitaceae family has long been considered a recalcitrant crop to induct hairy roots [20]. At present, only one article has reported obtaining *T. hemsleyanum* hairy roots via an *A. rhizogenes*-mediated approach [7]. In this report, hairy roots were induced using cucumopine type *A. rhizogenes* K599, and the induction and co-culture medium were optimized. These large-scale studies have provided valuable data to the scientific community exploring the *T. hemsleyanum* hairy root induction system. Nevertheless, due to the diversity in *A. rhizogenes* genotypes, explant type, infective mode, co-culture time, and induction medium type, it is difficult to formulate an integrative and comparative overview of the *T. hemsleyanum* hairy root induction system. Therefore, by optimizing the abovementioned factors, we established a high-efficiency agropine type *A. rhizogenes* Ar Qual-mediated hairy root induction system for *T. hemsleyanum*. Moreover, the *A. rhizogenes* Ar Qual strain carrying pCAMBIA 1381Z was used to mediate the expression of the exogenous gene GUS in transgenic hairy roots to verify the feasibility of the *A. rhizogenes* Ar Qual-mediated genetic transformation system.

Several factors affect hairy root induction, including the explant type, choice of *A. rhizogenes* strains, infective mode, co-cultivation period, and type of induction medium [21]. The sites that are most susceptible to *A. rhizogenes* infection during hairy root formation vary among species. For example, internodal explants of *Codonopsis pilosula* [21] had the highest induction rate of hairy roots, whereas leaf explants of *Momordica dioica* [22] induced hairy roots at a higher frequency. In this study, the leaf explants were highly sensitive to *A. rhizogenes* Ar Qual, with an induction rate 1 of 75.17 ± 7.42% and a positive rate of 96.57 ± 1.72%. Co-cultivation is an important stage for the transformation and integration of *A. rhizogenes* T-DNA into the host plant genome. When the co-cultivation time is too short, the infection efficiency is low, while an overgrowth of *A. rhizogenes* can be toxic to explants if the co-cultivation time is too long. In this study, a 3-day-long co-cultivation period yielded the highest induction rate 1 (65.14 ± 14.83%). In *C. pilosula*, the induction efficiency was the highest without co-cultivation, and decreased with the increase in co-cultivation days [21]. Du et al. [7] selected the optimal hormone content of 1.0 mg/L IBA and 1.0 mg/L KT using MS and B5 as basic media. Different media containing different nutrient elements may promote or inhibit hairy root induction. In their study, the induction effect of 1/4 MS nutrient medium supplemented with 1.0 mg/L IBA and 1.0 mg/L KT was optimal; this was followed by the 1/2 MS nutrient medium, while the induction effects of the MS and N6 media were the lowest. This is because the induction efficiency of *T. hemsleyanum* hairy roots varies with the nitrogen form and ratio, and with the salt ion concentration within different media. However, the effects of nitrogen form and ratio, and salt concentration, on the induction and growth of *T. hemsleyanum* hairy roots, as well as on metabolite biosynthesis, have not yet been reported. Consequently, the mechanism behind this requires further study.

### 3.2. High (+)-Catechin, (−)-Epicatechin, Luteolin-6-C-Glucoside, and Orientin Contents of T. Hemsleyanum Hairy Roots Induced by Agropine Type A. Rhizogenes Ar Qual

The hairy roots contained different secondary metabolites due to different Ri plasmid types of *A. rhizogenes*. Hairy roots induced using cucumopine type *A. rhizogenes* K599 were rich in kaempferol and quercetin [7], whereas hairy root induction systems for (+)-catechin and (−)-epicatechin, which are the active components in *T. hemsleyanum*, were hairless. In the existing hairy root systems that are rich in (+)-catechin and (−)-epicatechin, Ri plasmid types of *A. rhizogenes* were mostly agropine type *A. rhizogenes*; for instance, A4 [23], R1000 [24], and LBA9402 [25]. However, there is no report on the secondary metabolites in hairy roots induced using agropine type *A. rhizogenes* Ar Qual. In this study, the highest (+)-catechin and (−)-epicatechin contents in *T. hemsleyanum* hairy roots induced using *A. rhizogenes* Ar Qual were 692.63 ± 127.24 and 163.34 ± 31.86 μg/g DW, respectively.

Surprisingly, luteolin-6-C-glucoside is a luteolin derivative [26] that has many health-promoting benefits such as antioxidation and anti-inflammation [27]. Orientin, a flavonoid component with antioxidant properties, has been regarded as a promising nutraceutical for patients with liver damage [28]. Presently, they are mainly extracted from plants in a process characterized by high cost, low content, and long growth cycle. Luteolin-6-C-glucoside and orientin were detected in the six *T. hemsleyanum* hairy root lines. This is the first time that a hairy root system is established that can be used to study the biosynthetic mechanism and product of luteolin-6-C-glucoside and orientin.

The hairy roots were identified using *Rol B*, which can affect the formation and growth of hairy roots and activate metabolic defence signals to promote the synthesis and accumulation of metabolites [29]. Due to the uncertainty and heterogeneity of integrating T-DNA into plant genomes, there are often great differences in the growth rates and secondary metabolite synthesis of induced hairy roots [10]. Therefore, the six hairy root lines were different in morphology, biomass, and flavonoids content. HR13, with its fast growth rate and high flavonoids content (including (+)-catechin, (−)-epicatechin, neochlorogenic acid, luteolin-6-C-glucoside, and orientin) was selected to be compared with the true roots.

### 3.3. Higher Flavonoids Contents and Antioxidant Activity of T. hemsleyanum Hairy Roots Than True Roots

In this study, the IC50 of (+)-catechin was significantly higher than the IC50 of HR and TR, and the (+)-catechin content in hairy roots was significantly higher than that in true roots. Possibly, the difference in catechin yield is one of the reasons for the difference in antioxidative activity. Previously, statistical analysis showed a correlation between DPPH activity and catechin content (r = 0.7348) [30], which was consistent with the experimental results. Rigling et al. [31] tested the catechin profile using ultra-high-performance liquid chromatography tandem mass spectrometry (UHLPC-MS). The antioxidant capacity of catechin before and after fermentation was also tested using DPPH free-radical-scavenging activity, thereby proving the antioxidant activity of catechin. This supported the usefulness of *T. hemsleyanum* hairy roots with the same potential as *T. hemsleyanum* true roots for the production of valuable and naturally derived phytochemicals, such as (+)-catechin.

In conclusion, we further optimized the induction system of *T. hemsleyanum* hairy roots. Notably, the culture time was significantly shortened; the number of environmental requirements was reduced; and the biomass and flavonoids contents, especially the contents of (+)-catechin and (−)-epicatechin, were increased. Moreover, it is noteworthy to mention that this is the first time that a hairy root system was established that can be used to study the biosynthetic mechanism and product of luteolin-6-C-glucoside and orientin. The antioxidant activity of the hairy roots was higher than that of the true roots. Therefore, the hairy root induction system established in this study can be used as an effective alternative to true root production, and hairy roots can be regarded as a potential source of natural antioxidants. In addition, the study provides an effective alternative method to produce flavonoids as well as a good platform for exploring the regulatory pathway using the efficient expression of exogenous GUS protein.

## 4. Materials and Methods

### 4.1. Plant Materials and Chemicals

Sterile *T. hemsleyanum* seedlings were provided by Shanghai Xiancaotang Biotechnology Co., Ltd (Shanghai, China). and identified by Professor Wu Xueqian of Zhejiang A&F University.

The (+)-Catechin (CAS Registry Number 154-23-4), (−)-epicatechin (CAS Registry Number 490-46-0), neochlorogenic acid (CAS Registry Number 906-33-2), luteolin-6-C-glucoside (CAS Registry Number 4261-42-1), orientin (CAS Registry Number 28608-75-5), rutin (CAS Registry Number 153-18-4), 1,1-diphenyl-2-picrylhyrdrazyl (CAS Registry Number 1898-66-4), N6-benzyl adenine (CAS Registry Number 1214-39-7), acetosyringone (CAS Registry Number 2478-38-8), 1-naphthylacetic acid (CAS Registry Number 86-87-3), and 3-indolebutyric acid (CAS Registry Number 60096-23-3) were purchased from Shanghai Yuanye Bio-Technology Co., Ltd. (Shanghai, China). All standards were identified with the purity higher than 98%. Acetonitrile, methanol, and formic acid of UPLC grade were supplied by Anhui Tedia High Purity Solvents Co., Ltd. (Anhui, China). Sodium nitrite (CAS Registry Number 7632-00-0), aluminum nitrate (CAS Registry Number 7784-27-2), and sodium hydroxide (CAS Registry Number 1310-73-2) were bought from Sinopharm Chemical Reagent Co., Ltd. (Shanghai, China).

The subculture was initiated from the internodes of tissue culture plantlets of Zhejiang origin. The explants were micropropagated using MS nutrient medium supplemented with 0.8 mg/L N6-benzyl adenine (6-BA). For genetic transformation, seven-week-old and above, morphologically identical plants (5–7 cm) were used.

### 4.2. Induction of Hairy Roots

The induction of hairy roots was divided into the steps of strain activation and expansion, pre-culture, infection, co-cultivation, and induction. *Agrobacterium rhizogenes* (*A. rhizogenes*) C58C1 was activated and expanded under the culture conditions described by Naeini et al. [32]. The *A. rhizogenes* Ar Qual and *A. rhizogenes* Ar Qual harboring the binary vector pCAMBIA 1381Z (CAMBIA)—which contains the GUS open reading frame (ORF) and the selective marker gene kanamycin resistance (KAN) under the control of CaMV35S promoter—were activated and expanded under the culture conditions described by Su et al. [33]. Negative controls were set under the same conditions. The expanded bacterial suspension was poured into a 50 mL centrifuge tube and then centrifuged (RD-50DTZ, BIORIDGE) at 4100 rcf for 8 min. The supernatant was discarded, and the precipitate was dissolved and diluted in MS liquid medium (pH 5.8) supplemented with 0.4 mg/L 1-naphthylacetic acid (NAA) and 19.62 mg/L acetosyringone (AS). The optical density of the bacterial suspension was obtained with a spectrophotometer at OD600 = 0.5–0.6 of *A. rhizogenes* C58C1 and *A. rhizogenes* Ar Qual for genetic transformation.

As explants, young leaves or stems were excised from in vitro-grown plants and then cultivated in agar solidified MS medium (pH 5.8) supplemented with 0.4 mg/L NAA and 19.62 mg/L AS. The pre-culture continued from 24 h at 26 ± 0.2 °C. Then, the explants were wounded in the leaf veins or stems with a sterile scalpel, immersed for 5 min in the MS liquid medium (pH 5.8) supplemented with 0.4 mg/L NAA and 19.62 mg/L AS at OD600 = 0.5–0.6 of *A. rhizogenes* C58C1 and *A. rhizogenes* Ar Qual, respectively. According to whether to vacuum, the plant material was infected using different methods. All infected leaves or stems were transferred onto MS solid medium supplemented with 0.4 mg/L NAA and 19.62 mg/L AS. The co-cultivation continued from 1 to 4 days at 26 ± 0.2 °C in the dark. After co-cultivation, the explants were transferred onto induction medium containing 400, 350, or 300 mg/L carbenicillin disodium salt to remove the bacteria. The influence of different co-cultivation durations (1, 2, 3, and 4 days) and induction medium types (MS, 1/2 MS, 1/4 MS, B5, and N6 solid medium supplemented with 1.0 mg/L 3-indolebutyric acid (IBA), and 1.0 mg/L 6-Furfurylamino-purine (KT)) was investigated to optimize the conditions for gene transfer (Supplementary Materials Table S1). The transformation frequency (expressed as the percentage of inoculated explants producing roots) was recorded after 15 days. ‘Induction rate 1’ was defined as the percentage of explants induced by *Agrobacterium rhizogenes* to produce hairy roots out of the total number of explants infected. ‘Induction rate 2’ was calculated as the number of hairy roots induced with and without the presence of *Rol B* fragments divided by the total number of explants infected by *Agrobacterium rhizogenes*.

### 4.3. Identification of Hairy Roots

Genomic DNA was extracted from the hairy roots and subjected to polymerase chain reaction (PCR) with the MightyAmp^TM^ Genotyping Kit in accordance with the manufacturer’s instructions (TaKaRa). The genomic DNA from the roots of non-transformed control plants was used as the negative control. The PCR results were used to confirm the presence of the *Rol* B and *KAN* genes in the roots. The A∼678-bp fragment of the *Rol* B gene was amplified using the primer pair 5′-GATATATGCCAAATTTACACTAG-3′ and 5′-GTTAACAAAGTAGGAAACAGG-3′. The A∼455-bp fragment of the *KAN* gene was amplified using the primer pair 5′-TCGGCTATGACTGGGCACAACA-3′ and 5′-TCGGCAGGAGCAAGGTGAGATG-3′. The PCR was carried out using the following cycle conditions: pre-denaturation at 94 °C for 4 min, followed by 5 cycles of 10 s denaturation at 98 °C, 15 s annealing at 60–50 °C cutting down 2 °C per cycle for the amplification of the *Rol* B and KAN fragments and 1 min extension at 68 °C, 25 cycles of 10 s denaturation at 98 °C, 15 s annealing at 58 °C for the amplification of the *Rol* B and *KAN* fragment and 1 min extension at 68 °C. The mixture was amplified in a T100™ Thermal Cycler (BIO-RAD, USA). The amplified DNAs were mixed with 5 × loading Dye at a ratio of 1:4 for staining, detected on 1.0% (*w*/*v*) agarose gels in 1 × TAE buffer at 150 V, and then visualized by Imagemaster VDS. ‘Positive rate’ was defined as the proportion of hairy roots that contained *Rol B* fragments among all the hairy roots with or without *Rol B* fragments. ‘Positive acquisition rate’ was obtained by multiplying the ‘positive rate’ by ‘induction rate 2’.

The histochemical assay for the reporter gene GUS activity was performed using a GUS histochemical assay kit (Real-Times) following the manufacturer’s protocol. Then the reaction mixture was placed under mild vacuum for 5 min and incubated overnight at 37 °C. Staining of the hairy roots was observed and captured by a digital camera.

### 4.4. Selection of Hairy Root Lines with High Biomass and High Flavonoids Production

Healthy hairy root lines 5, 12, 13, 14, 16, and 20 (200 mg fresh mass) were taken for liquid culture. The B5 liquid medium was supplemented with 1.0 mg/L KT and 1.0 mg/L IBA (pH 5.5). The cultures were stored under continuous agitation at 130 rpm in an orbital shaker and incubated at 26 ± 1 °C in the dark. The biomass of hairy roots was assessed at 30 days of culture. The roots were separated from the media and washed with distilled water, and excess surface water was blotted away. The samples were quick-frozen with liquid nitrogen for 10 min, frozen at −80 °C for 24h, and then dried at −5 °C and 15 Pa for 48 h with a freeze-dryer (FD-1A-50, Shanghai Hefan). Their DW was determined later.

The content of total flavonoids was determined by the colorimetric method [34] with some modifications. Lyophilized samples of hairy root lines 5, 12, 13, 14, 16, and 20 were ground into powder. Then, 0.1 g of the freeze-dried powder of hairy roots was ultrasonically extracted with 5 mL 70% methanol for 90 min and centrifuged at 4100 rcf for 10 min. The supernatant of the hairy roots was the solution to be tested. In addition, 240 μL of the extract was placed in a 2 mL centrifuge tube, added with 40 μL of 5% sodium nitrite, shaken well, allowed to stand for 6 min, added with 40 μL of 10% aluminum nitrate, shaken well, allowed to stand for 6 min, added with 400 μL 1 mol/L sodium hydroxide, shaken well, added with 0.28 mL of water to 1 mL, and then allowed to stand for 15 min. Absorbance was obtained at 500 nm. All the experiments were performed in triplicate with at least three independent runs (*n* = 9).

Next, 0.1 g of the freeze-dried powder of six *T. hemsleyanum* hairy root lines was ultrasonically extracted with 5 mL 70% ethanol for 90 min and centrifuged at 4100 rcf for 10 min. The supernatants were prepared by being passed through a 0.22 µm PTFE filter membrane, and then placed in a sample bottle for high-performance liquid chromatography (HPLC) to analyze the contents of (+)-catechin and (−)-epicatechin. The Waters 2690 binary high-performance liquid chromatograph was used for content determination, and the detector was 2998 PDA. The Waters Sunfire C18 column (250 mm × 4.6 mm, 5 μm) was used. Data were collected using the data acquisition software Empower2. The liquid phase conditions were as follows: (1) Chromatographic column: Waters Sunfire C18 column (250 mm × 4.6 mm, 5 μm); (2) Mobile phase: Phase A, acetonitrile (acetonitrile: methanol: formic acid = 950:50:1) and Phase B, water (ultra-pure water: methanol: formic acid = 950:50:1); (3) Gradient elution program:0 min A/B is 10:90 (*v*/*v*), 25 min A/B is 20:80 (*v*/*v*), 45 min A/B is 30:70 (*v*/*v*), 50 min A/B is 80:20 (*v*/*v*), 56 min A/B is 10:90 (*v*/*v*); and (4) flow rate of 1.0 mL/min, column temperature of 25 °C, and injection volume of 40 µL. The detection wavelength was set at 278 nm. All the experiments were performed in triplicate with at least three independent runs (*n* = 9).

Then, 0.5 g of the freeze-dried powder of six *T. hemsleyanum* hairy root lines was ultrasonically extracted with 10 mL of 70% ethanol for 30 min and centrifuged at 4100 rcf for 10 min. Repeat twice. Rotate the supernatant at 50 °C and 70 rpm and dissolve it with 3.5 mL 70% methanol. The above samples were prepared by being passed through a 0.22 µm PTFE filter membrane, and then placed in a sample bottle for HPLC to analyze the contents of neochlorogenic acid, luteolin-6-C-glucoside, and orientin. Mobile phase A is acetonitrile and Mobile phase B is water (ultra-pure water: methanol: formic acid = 950:50:1). Injection volume is 10 µL. The detection wavelength was set at 330 nm. Gradient elution program:0 min A/B is 10:90 (*v*/*v*); 30 min A/B is 17:83 (*v*/*v*); 60 min A/B is 20:80 (*v*/*v*); 65 min A/B is 10:90 (*v*/*v*); flow rate of 1.0 mL/min; column temperature of 25 °C. All the experiments were performed in triplicate with at least three independent runs (*n* = 9).

### 4.5. Comparison of Flavonoids in Hairy Roots and True Roots

Lyophilized samples of hairy roots line HR13 and true roots were ground into powder to compare total flavonoids, (+)-catechin, (−)-epicatechin, neochlorogenic acid, luteolin-6-C-glucoside, and orientin contents. The total flavonoids contents of HR13 and true roots were determined following the method described in determination of total flavonoids in different hairy root lines. The (+)-catechin, (−)-epicatechin, neochlorogenic acid, luteolin-6-C-glucoside, and orientin contents of HR13 and true roots were determined following the method described in different hairy root lines.

### 4.6. Measurement of 1,1-Diphenyl-2-picrylhyrdrazyl Free-Radical Scavenging Activity

The 1,1-diphenyl-2-picrylhyrdrazyl (DPPH) free-radical scavenging activity was measured as previously described by Tai et al. [35], with slight modifications. A 0.2 mM solution of DPPH was prepared in methanol. Hairy roots line HR13 extract solution and true roots extract solution, respectively, were prepared by 0.1 g of the freeze-dried powder ultrasonically extracted with 5 mL 70% methanol for 90 min and centrifuged at 4100 rcf for 10 min. Then, the supernatant of the hairy roots and the supernatant of the true roots were shaken up as the solution to be tested. Different volumes of the solution (0.02, 0.04, 0.06, 0.08, 0.10, 0.12, 0.14, 0.16, 0.18, and 0.20 mL) were assayed in 2 mL centrifuge tubes, added with 70% ethanol to 0.20 mL, and then blended. Next, 0.30 mL of DPPH solution was added and then mixed. The mixture was placed in the dark for 30 min, and experiments were repeated three times. The DPPH free-radical scavenging activity was calculated as an inhibition percentage based on the following equation: Inhibition (%) = [(A0 − A1)/A0] × 100, where A0 is the absorbance of the control and A1 is the absorbance of the sample. The IC50 values of HR and TR are indicative of the content of flavonoids from Section 4.5.

### 4.7. Statistical Analysis

Data used to determine transformation frequency were collected from three separate experiments and presented as mean values ± standard error. Data were subjected to one-way ANOVA for comparison of means, and significant differences were calculated using Fisher’s LSD test at the 5% level with a statistical software package (Graphpad Prism 8 for Windows).

## 5. Conclusions

In conclusion, we further optimized the induction system of *T. hemsleyanum* hairy roots. Substantially, the culture time was significantly shortened, environmental requirements were reduced, and the biomass and flavonoids contents were increased, especially (+)-catechin and (−)-epicatechin. Moreover, it is noteworthy that this is the first time that a hairy roots system has been established that can be used to study the biosynthetic mechanism and product of luteolin-6-C-glucoside and orientin. The antioxidant activity of the hairy roots was higher than that of the true roots. Therefore, the hairy root induction system established in this study can be used as an effective alternative for true roots production, and hairy roots can be regarded as a potential source of natural antioxidants. In addition, the study offers an effective alternative method to produce flavonoids and provides a good platform for exploring the regulatory pathway by the efficient expression of exogenous GUS protein.

## Figures and Tables

**Table 1:** Hairy roots induction from leaves and stems explants by *A. rhizogenes* C58C1 and Ar Qual.

*A. rhizogenes*	Explant Type	Induction Rate 1 (%)	Induction Rate 2 (%)	Positive Rate (%)	Positive Acquisition Rate (%)
C58C1	stems	90.80 ± 2.81 ^a^	337.90 ± 15.80 ^a^	5.43 ± 0.70 ^a^	18.14 ± 1.47 ^a^
C58C1	leaves	36.73 ± 4.02 ^b^	101.23 ± 42.52 ^b^	39.40 ± 0.80 ^b^	40.00 ± 17.14 ^b^
Ar Qual	stems	90.48 ± 9.24 ^a^	342.86 ± 47.72 ^a^	88.15 ± 3.45 ^c^	25.71 ± 13.42 ^ab^
Ar Qual	leaves	75.17 ± 7.42 ^c^	426.67 ± 52.49 ^c^	96.57 ± 1.72 ^d^	317.27 ± 57.10 ^d^

Significant differences in the same column are represented by different letters (a–d) (*p* < 0.05).

**Table 2:** Effect of infective mode on induction.

Infective Mode	Induction Rate 1 (%)	Induction Rate 2 (%)
No vacuum	79.37 ± 9.64 ^a^	396.45 ± 74.65 ^a^
Vacuum (0.5 min)	38.33 ± 3.67 ^b^	90.32 ± 4.92 ^b^
Vacuum (1.0 min)	16.90 ± 5.97 ^c^	20.13 ± 18.67 ^c^

Significant differences in the same column are represented by different letters (a–c) (*p* < 0.05).

**Table 3:** Effect of co-culture times on induction.

Co-cultivation Times	Induction Rate 1 (%)	Induction Rate 2 (%)
1 d	5.30 ± 1.33 ^a^	5.30 ± 5.22 ^a^
2 d	18.76 ± 5.59 ^b^	82.14 ± 21.71 ^b^
3 d	65.14 ± 14.83 ^c^	326.65 ± 64.75 ^c^
4 d	8.70 ± 3.83 ^a,b^	26.65 ± 10.90 ^d^

Significant differences in the same column are represented by different letters (a–d) *(p* < 0.05).

**Table 4:** Effect of induction medium types on induction.

Induction Medium Types	Induction Rate 1 (%)	Induction Rate 2 (%)
MS	25.00 ± 8.33 ^a^	52.78 ± 31.55 ^a^
1/2 MS	69.05 ± 27.04 ^b^	382.14 ± 93.55 ^b^
1/4 MS	95.83 ± 7.22 ^b^	951.39 ± 231.85 ^c^
B5	60.17 ± 12.85 ^a,b^	326.65 ± 90.89 ^b^
N6	16.67 ± 5.56 ^a^	16.67 ± 5.56 ^a^

Significant differences in the same column are represented by different letters (a–c) (*p* < 0.05).

**Table 5:** Standard curves, LOD, and LOQ were established by HPLC.

NO.	Component Name	Linear	R^2^	Linear Range (μg/mL)	LOD (μg/mL)	LOQ (μg/mL)
1	(+)-Catechin	y = 36,373x − 5711.7	0.9993	40.00–240.00	0.150	0.280
2	(−)-Epicatechin	y = 25,331x + 1938.2	0.9990	0.30–24.00	0.150	0.300
3	Neochlorogenic acid	y = 17,879x − 18,429	0.9992	1.23–66.67	0.025	0.063
4	Luteolin-6-C-glucoside	y = 33,118x − 67,899	0.9997	1.23–66.67	0.029	0.060
5	Orientin	y = 15,053x – 36,659	0.9992	1.23–66.67	0.079	0.200

**Table 6:** Flavonoids contents of hairy roots and true roots.

Samples	The Content of Each Compound (μg/g DW) *					
	Total Flavonoids	(+)-Catechin	(−)-Epicatechin	Neochlorogenic Acid	Luteolin-6-C-Glucoside	Orientin
TR	19,851.63 ± 575.36 ^a^	622.52 ± 97.53 ^a^	70.21 ± 25.12 ^b^	15.23 ± 0.38 ^b^	185.29 ± 1.19 ^b^	44.06 ± 0.79 ^b^
HR	8919.48 ± 740.97 ^b^	692.63 ± 127.24 ^a^	163.34 ± 31.86 ^a^	45.95 ± 3.46 ^a^	209.68 ± 6.03 ^a^	56.82 ± 4.75 ^a^

* Mean ± SDs not sharing the same lowercase letters are significantly different (*p* < 0.05), *n* = 9.

**Table 7:** Results of DPPH free-radical scavenging assay.

No.	Name	IC50 (μg/mL) *
1	HR	1.64 ± 0.16 ^f^
2	TR	2.34 ± 0.15 ^e^
3	Rutin	3.29 ± 0.54 ^d^
4	(+)-Catechin	1.41 ± 0.24 ^f^
5	Orientin	23.10 ± 6.67 ^b^
6	Luteolin-6-C-glucoside	20.32 ± 0.32 ^b^
7	Neochlorogenic acid	50.39 ± 4.4 ^a^
8	(−)-Epicatechin	10.46 ± 2.49 ^c^

* Mean ± SDs not sharing the same lowercase letters are significantly different (*p* < 0.05), *n* = 9.

## Data Availability

The datasets used and/or analyzed during the current study are available from the corresponding author on reasonable request.

## Supplementary Materials

The following supporting information can be downloaded online at https://www.mdpi.com/article/10.3390/molecules28062686/s1. Figure S1: (+)-Catechin and (−)-epicatechin contents of *T. hemsleyanum* hairy root lines as determined by HPLC. Figure S2: Neochlorogenic acid contents of *T. hemsleyanum* hairy root lines as determined by HPLC. Figure S3: Luteolin-6-C-glucoside and orientin contents of *T. hemsleyanum* hairy root lines as determined by HPLC. Figure S4: Flavonoids contents of hairy roots (HR) and true roots (TR). Table S1: The chemical composition of MS, 1/2 MS, 1/4 MS, B5, and N6 medium.

## Abbreviations

*T. hemsleyanum*	*Tetrastigma hemsleyanum* Diels et Gilg
*A. rhizogenes*	*Agrobacterium rhizogenes*
HPLC	high-performance liquid chromatography
DW	dry weight
6-BA	N6-benzyl adenine
AS	acetosyringone
NAA	1-naphthylacetic acid
IBA	3-indolebutyric acid
KT	6-Furfurylamino-purine
ORF	open reading frame
KAN	kanamycin resistance
PCR	polymerase chain reaction
LOD	limit of detection
LOQ	limit of quantitation
DPPH	1,1-diphenyl-2-picrylhyrdrazyl
HR	hairy root
TR	true root
UHLPC-MS	ultra-high-performance liquid chromatography tandem mass spectrometry
