# Supplementary material for: Induction, Flavonoids Contents, and Bioactivities Analysis of Hairy Roots and True Roots of Tetrastigma hemsleyanum Diels et Gilg
Source: Molecules. 2023 Mar 16;28(6):2686. doi: 10.3390/molecules28062686 (PMC10053805; doi:10.3390/molecules28062686)
Supplement: Supplementary file 1 [file molecules-28-02686-s001.zip › molecules-2207660-supplementary.pdf]

Supplementary Materials

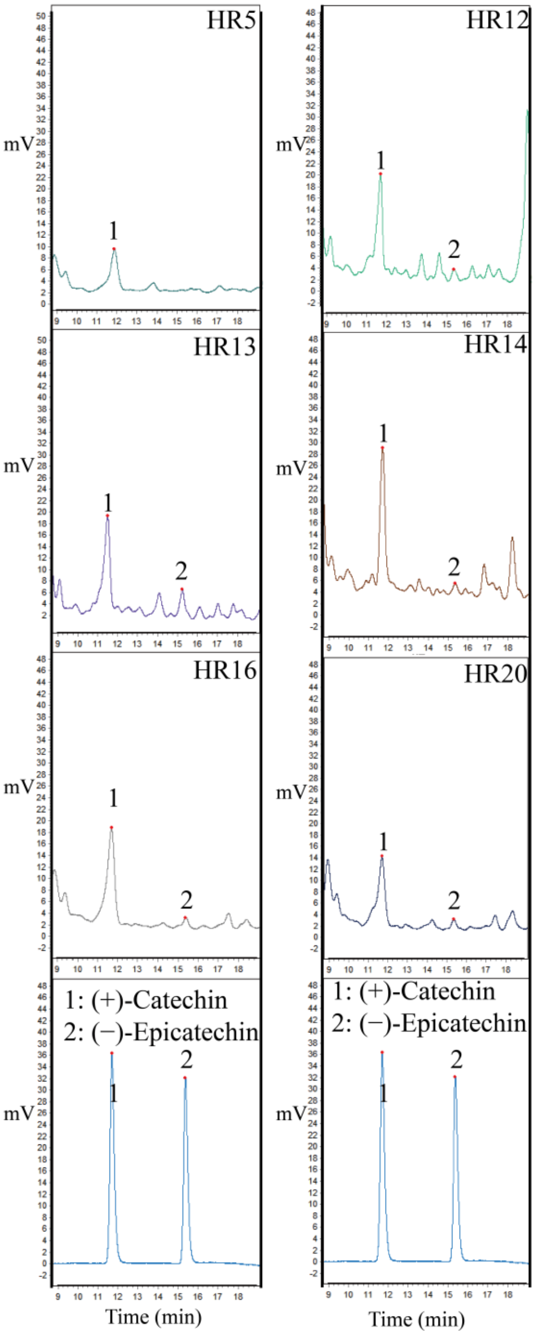

Figure S1. (+)-Catechin and (−)-epicatechin contents of *T. hemsleyanum* hairy root lines as determined by HPLC. PubChem CID of (+)-catechin (1) is 9064. PubChem CID of (−)-epicatechin (2) is 72276.

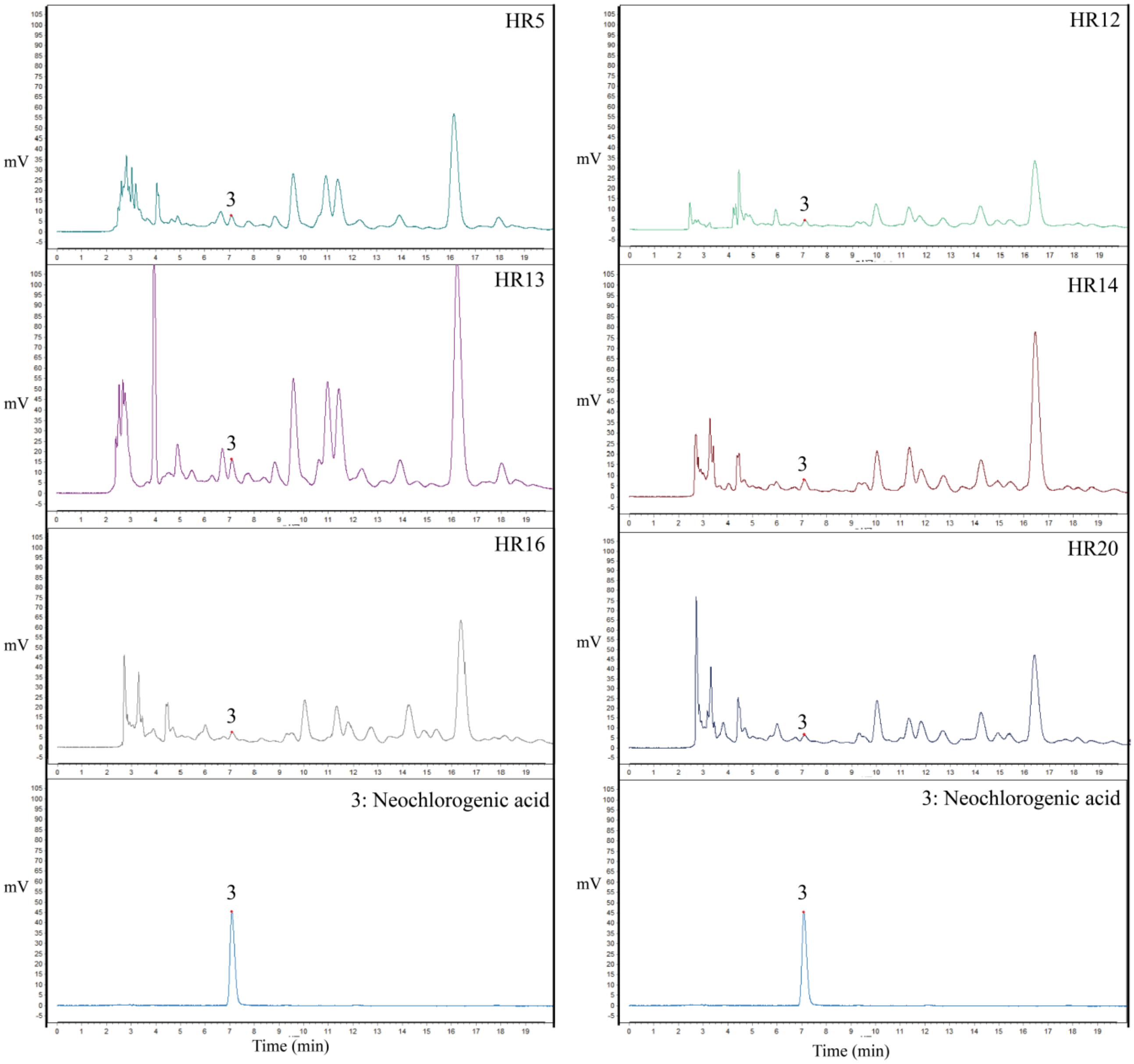

Figure S2. Neochlorogenic acid contents of *T. hemsleyanum* hairy root lines as determined by HPLC. PubChem CID of neochlorogenic acid (3) is 5280633.

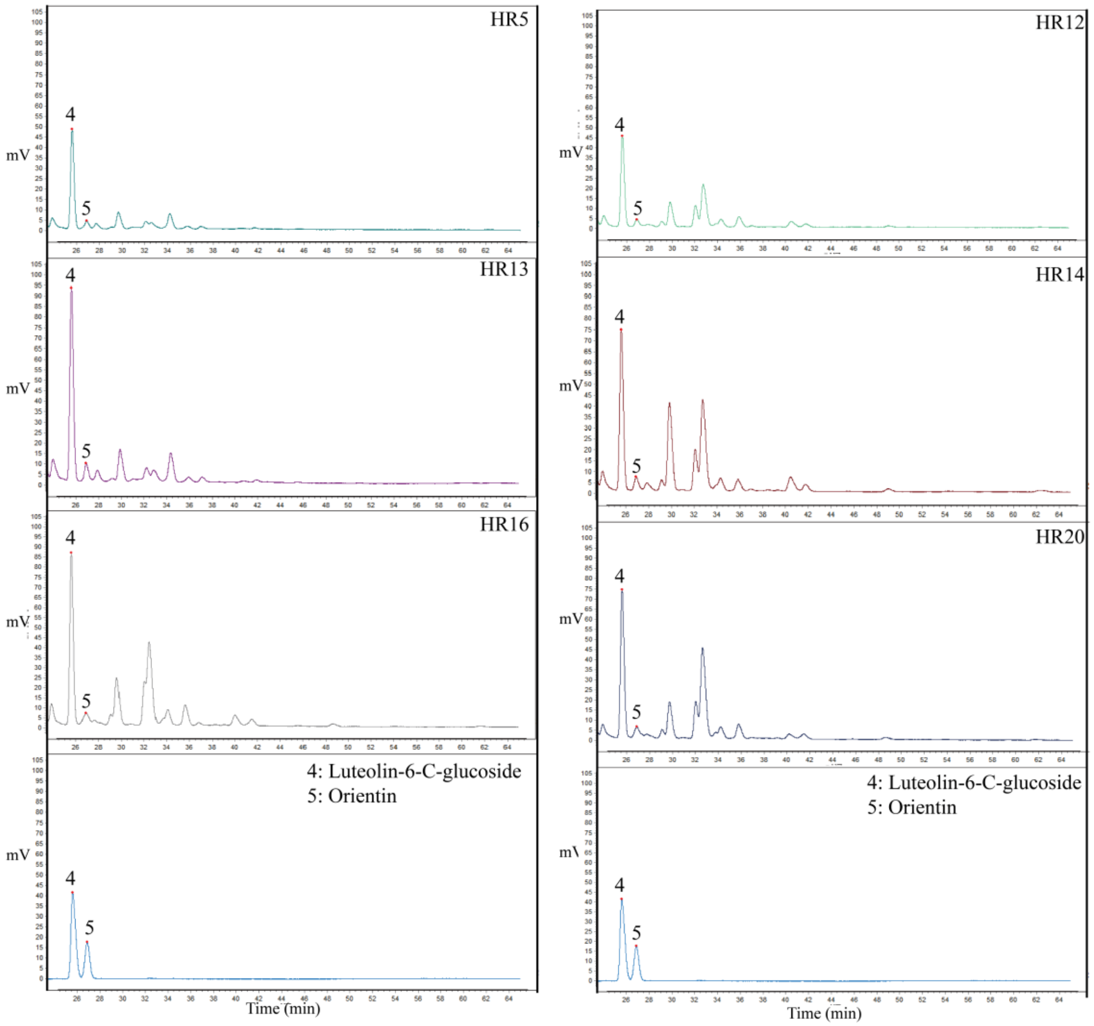

Figure S3. Luteolin-6-C-glucoside and orientin contents of *T. hemsleyanum* hairy root lines as determined by HPLC. PubChem CID of luteolin-6-C-glucoside (4) is 114776. PubChem CID of orientin (5) is 5281675.

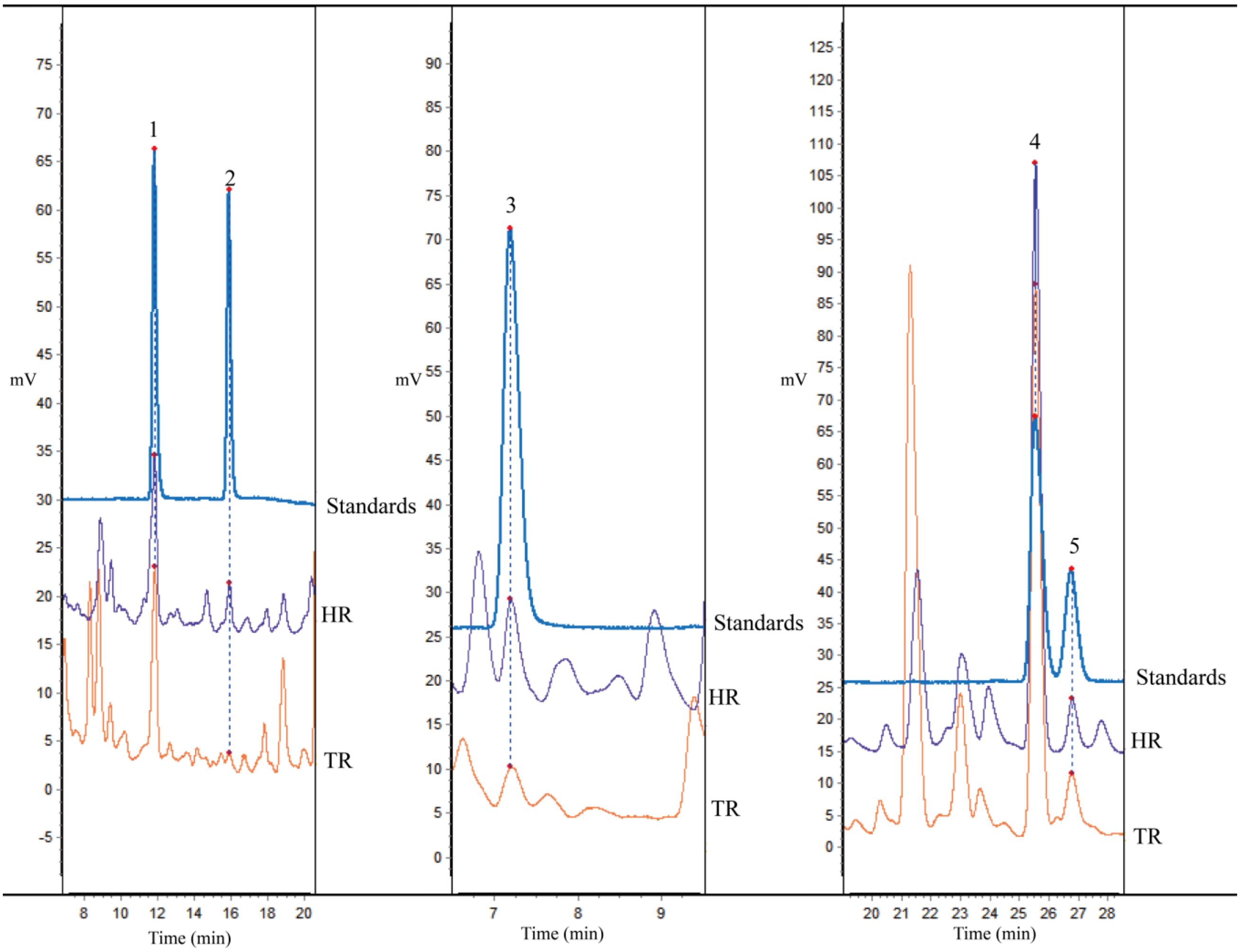

Figure S4. Flavonoids contents of hairy roots (HR) and true roots (TR). PubChem CID of (+)-catechin (1) is 9064. PubChem CID of (−)-epicatechin (2) is 72276. PubChem CID of neochlorogenic acid (3) is 5280633. PubChem CID of luteolin-6-C-glucoside (4) is 114776. PubChem CID of orientin (5) is 5281675.

Table S1. The chemical composition of MS, 1/2 MS, 1/4 MS, B5, and N6 medium.

Chemical composition name	The Content of Each Compound (mg/L)				
	MS	1/2 MS	1/4 MS	B5	N6
Potassium Nitrate	1900.00	950.00	475.00	2500.00	2800.00
Ammonium Nitrate	1650.00	825.00	412.50	0.00	463.00
Potassium Phosphate Monobasic	170.00	85.00	42.50	150.00	400.00
Magnesium Sulfate	370.00	185.00	92.50	122.09	185.00
Calcium Chloride	440.00	220.00	110.00	113.24.00	165.00
Potassium Iodide	0.83	0.83	0.83	0.75	0.80
Boric Acid	6.20	6.20	6.20	3.00	1.60
Manganese Sulfate	22.3	22.3	22.3	10	4.4
Zinc Sulfate	8.60	8.60	8.60	2.00	1.50
Sodium Molybdate	0.25	0.25	0.25	0.25	0.00
Cupric Sulfate	0.025	0.025	0.025	0.025	0.00
Cobalt Chloride·$6H_2O$	0.025	0.025	0.025	0.025	0.00
Na_2EDTA	37.30	37.30	37.30	37.30	37.30
Ferrous Sulfate	27.80	27.80	27.80	27.80	27.80
Myo-Inositol	100.00	100.00	100.00	100.00	0.00
Glycin	2.00	2.00	2.00	0.00	2.00
Thiamine·HCL	0.10	0.10	0.10	10.00	1.00
Pyridoxine·HCL	0.50	0.50	0.50	1.00	0.50
Nicotinic Acid	0.50	0.50	0.50	1.00	0.50
Ammonium Sulfate	0.00	0.00	0.00	134.00	0.00
